# Antibacterial activity of fig leaf (*Ficus carica* Linn.) extract against *Enterococcus faecalis* and its cytotoxicity effects on fibroblast cells

**DOI:** 10.14202/vetworld.2018.342-347

**Published:** 2018-03-20

**Authors:** Intan Nirwana, Devi Rianti, R. Helal Soekartono, Rr. Dwi Listyorini, Desi Putri Basuki

**Affiliations:** 1 Department of Dental Materials, Faculty of Dental Medicine, Universitas Airlangga, Surabaya, Indonesia; 2 Undergraduate Student of Faculty of Dental Medicine, Universitas Airlangga, Surabaya, Indonesia

**Keywords:** *Enterococcus faecalis*, fig leaf (*Ficus carica* Linn.) extract, minimum bactericidal concentration, 3-(4,5-dimethylthiazol-2-yl)-2,5-diphenyltetrazolium bromide assay

## Abstract

**Background:**

*Enterococcus faecalis* is one of the bacteria that commonly found in root canal and pulp infection after root canal treatment. Sodium hypochlorite is the most widely used root canal irrigation, but it has toxic properties if exposed to periradicular tissues. It is necessary to develop an alternative for root canal irrigation. Fig leaf (*Ficus carica* Linn.) extract contains active substances such as flavonoid, tannin, and terpenoid which have been known for their antibacterial potency.

**Aim:**

This study aimed to determine the minimum bactericidal concentration (MBC) of fig leaf (*F. carica* Linn.) extract against *E. faecalis* and its cytotoxicity on fibroblast cells *in vitro*.

**Materials and Methods:**

A serial dilution method was used to determine the MBC of fig leaf extract on *E. faecalis* which grown on nutrient agar media. Inoculation was carried out at concentrations that suspected minimum inhibitory concentration (MIC), MBC, concentration between MIC and MBC, and control groups on different nutrient agar. MIC and MBC of fig leaf extract against *E. faecalis* were known by counting the growth of bacteria colonies on nutrient agar media in CFU/ml. The cytotoxicity of MIC and MBC of the extract acquired were tested using 3**-**(4,5-dimethylthiazol-2-yl)-2,5-diphenyltetrazolium bromide assay, and the results were read using an ELISA reader. Data of *E. faecalis* colonies were analyzed using Kruskal–Wallis and Mann–Whitney test.

**Results:**

The result showed a significant difference among the groups (p<0.05). fig leaf extract at a concentration of 50% showed no bacterial growth, and cell viability at this concentration was 77.7%.

**Conclusion:**

Fig leaf extract has antibacterial effect on *E. faecalis* with MBC at 50% and not cytotoxic to fibroblast cells.

## Introduction

Root canal treatment in dentistry is a treatment option for pulp disease by removing bacteria and metabolic waste inside the root canal system. Root canal treatment, a procedure to clean and seal the root canal system, aims to remove pathogens, prevent reinfection, and allow healing process to take place [1]. Irrigation is the important step of root canal treatment . Irrigation material serves as a debris solvent and made the instrument movement easier during root canal preparation [2]. The ideal root canal irrigation material should have low surface tension and low toxicity as well as should be smear layer-free, inexpensive, and easy to use [3].

The most common bacteria found in root canals are *Enterococcus faecali*s. These bacteria are usually found in the fail root canal treatment. The prevalence of *E. faecalis* bacteria in root canal failure reached 38% [4].

The irrigation material that commonly used in root canal treatment is sodium hypochlorite, which has certain weaknesses, such as becoming toxic when in contact with periradicular tissue. Sodium hypochlorite is antimicrobial to bacteria, both Gram-positive and negative, spores, fungi, and viruses [5]. However, the optimal property of NaOCl to dissolve organic tissue is non-selective that means it is able to dissolve both necrotic and vital pulp remnants indistinguishably, especially at high concentrations.The *in vitro* studies showed that 0.5% sodium hypochlorite has a longer incubation time than other irrigation ingredients in eradicating *E. faecalis* [6].

fig leaf can be used as an alternative treatment due to its nature of *E. faecalis* resistance. The fig leaf contains several active compounds such as flavonoids, tannins, sesquiterpenes, alkaloids, and saponins [7] which have biological activities as antioxidant, anticancer, anti-inflammation, antiviral, and antibacterial [8]. As a candidate of root canal irrigation material, nevertheless, fig leaf extract must satisfy biocompatibility requirement, therefore the material applied on the host tissue does not cause damage or injury. Cytotoxicity assessment is a prerequisite for the materials biocompatibility evaluation [9-12]. Thus, the cytotoxicity of an agent can be defined as toxicological risks of a material or its extract in cell culture [11,13]. The interaction between the material and its components with the cell will cause tissue reactions, such as inflammation and necrosis [14]. Cytotoxicity assessment of *Ficus carica* Linn. leaf extract as a canal irrigation material is indispensable because of the close contact with gingiva and the oral mucosa connective tissue [9].

Moreover, the safety of a plant as a therapeutic agent candidate must be ensured and its side effects should be acceptable to the host. Bioactive compounds that have no or less toxic effect on the host can be considered as good candidates for drug formulations [15]. Therefore, cytotoxic levels of medicinal plants should also be evaluated against host cells.

*E. faecalis* is a bacterium that can colonize in the dentin tubules and survive in the root canal without the support of other bacteria [16]. Previous researches, however, still have not known the minimum bactericidal concentration (MBC) of the fig leaf extract for *E. faecalis*.

Based on the description above, it is necessary to perform a research on the activity of the fig leaf extract as an antibacterial agent against *E. faecalis* as an alternative to root canal irrigation materials. This research aimed to determine the MBC of fig leaf extract against *E. faecalis* and its cytotoxicity against fibroblast cell *in vitro*.

## Materials and Methods

### Ethical approval

All proceedings were approved by the Ethical Committee of Universitas Airlangga (number 248/KKEPK.FKG/X/2016).

### Research materials

The materials used to determine the MBC of fig leaf (*F. carica* Linn.) extract against *E. faecalis* were fig leaf extract, 96% ethanol, *E. faecalis* bacteria, Brain Heart Infusion Broth (BHIB), and nutrient agar (Oxoid CM0003).

The materials used for the MTT test were 3-(4,5-dimethylthiazol-2-yl)-2,5-diphenyltetrazolium bromide, 10% fetal bovine serum (FBS), 100 units/ml of fungizone, dimethyl sulfoxide (DMSO), human gingival fibroblast cells (primary culture), Eagle’s minimum essential medium (EMEM), kanamycin, and phosphate-buffered saline.

### Research methods

#### Preparation of plant material

Criteria of fig leaf were 4-6 months’ green Jordan types which could be picked approximately on the fifth leaf and so on from the top of the fig tree. fig leaf extract was prepared using maceration method with 96% ethanol solvent.

#### Phytochemical screening of fig leaf extract

In phytochemical screening, the following groups of active ingredients were surveyed: Flavonoids, terpenoids, saponins, tannins, and alkaloids.

Identification of flavonoids

About 1 ml of 10% lead acetate solution was added to 1 ml of extract. The formation of a yellow precipitate was taken as a positive test for the presence of flavonoids.

Identification of terpenoids

About 2 ml of the extract was dissolved in 2 ml of CHCl_3_ and evaporated to dryness. 2 ml of H_2_SO_4_ was then added and heated for about 2 min. Development of a grayish color indicates the presence of terpenoids.

Identification of saponins

Nearly 5 ml of extract was shaken vigorously with 5 ml of distilled water in a test tube and warmed. The formation of stable foam was taken as an indication of the presence of saponins.

Identification of tannins

About 2 ml of the extract was stirred with 2 ml of distilled water and few drops of ferric chloride (FeCl_3_) solution was added. Formation of green precipitate was an indication of the presence of tannins.

Identification of alkaloids

About 3 ml of extract was stirred with 3 ml of 1% HCl on steam bath. 1 ml of mixture was taken separately in two test tubes. Few drops of Dragendorff’s reagent were added in one tube and occurrence of orange–red precipitated was taken as positive. To the second tube, Mayer’s reagent was added and appearance of buff-colored precipitate was taken a positive test for the presence of alkaloids [17].

### Preparation of E. faecalis

*E. faecalis* was taken from the *E. faecalis* stock of microbiology laboratory, Faculty of Dental Medicine, Universitas Airlangga, using a sterile inoculating loop and put into a test tube containing the BHIB liquid medium. *E. faecalis* cultures were put into the anaerobic jar under an anaerobic atmosphere and then incubated in an incubator at a room temperature of 37°C for 24 h to observe its turbidity to be equalized to a standard of 0.5 McFarland (10^8^ CFU/ml).

### Minimum inhibitory concentration (MIC) and MBC determination

MIC is the lowest concentration of an antimicrobial agent that can inhibit the visible growth of a microorganism after incubated overnight [18]. Serial dilutions of the fig leaf extract were done in macrodilution tubes with the concentration of 100%, 50%, 25%, 12.5%, 6.25%, 3.125%, 1.565%, and 0.781%. *E. faecalis* suspensions that had been adjusted to the logarithmic-phase growth to match the turbidity of a 0.5 McFarland standard (10^8^ CFU/mL) were added to all tubes as much as 0.1 mL, and the tubes then were incubated at 37°C for 24 h.

Each tube was examined for bacterial growth and compared to the control. The positive control tube was filled with a standardized *E. faecalis* suspension with 0.5 McFarland (10^8^ CFU/mL) as much as 0.1 ml and BHIB medium. The negative control tube was filled with BHIB media. The result of dilution series of fig leaf extract on the growth of *E. faecalis* was characterized by the presence of turbidity or sediment. The absence of bacterial growth was defined as antibacterial activity.

MBC is the lowest concentration of the fig leaf extract required to kill a particular bacterium. Six dilutions were conducted in duplicate for the MBC test. After incubated for 24 h, the tubes were analyzed for MIC, and then, MBC was determined by sampling all the macroscopically clear tubes (1 dilution below the MIC was used for the levels to be assessed in the MBC assay) and the first turbid tube in the series. The suspension as much as 0.1 mL was inoculated onto the plates of nutrient agar (Oxoid CM0003). The plates were incubated for 24 h at 37°C. Each experiment was carried out 3 times and correlated against the control [19]. MIC and MBC obtained will be used for cytotoxicity test using MTT assay.

### In vitro cytotoxicity MTT assay

Cytotoxicity was assessed by MTT method. Human gingival fibroblast cells culture and microplates with 96 sterile wells were prepared in laminar flow. Wells on the first column of microplates were filled with EMEM, Kanamycin, 1% penstrep, 10% FBS, and 100 units/ml of fungizone as much as 100 μl used as control media. Wells on the second column of microplates were filled with fibroblast cells with a density of 3×10^3^ in EMEM, kanamycin, 1% penstrep, 10% FBS, and 100 units/ml of fungizone as much as 100 μl as control cell. The fig leaf extract with the MIC value of 37.5% and the MBC value of 50% was sterilized using UV sterilizer and was added to each well in the 3^rd^ and 4^th^ columns as much as 50 μl. Each treatment was repeated 10 times at a different well.

The microplates were incubated with 5% CO_2_ at 37°C for 20 h. The microplates then were removed from the incubation device, while culture medium and the fig leaf extract in those wells were taken with a syringe. Fibroblast cells, nevertheless, were still remained in the wells. Afterward, each well was refilled with 100 μl of culture medium. Twenty microliters MTT solution that had been filtered using a millipore of 0.20 μm was added to each well. The plates were further incubated for 4 h and the medium was removed. Fifty microliters of DMSO was added to each well to dissolve the formazan crystals. The optical density (OD) value of formazan was read by Elisa reader with a wavelength of 620 nm. To assess the percentage of cell viability, the following formula was used [20].


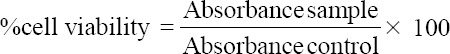


Absorbance control is the absorbance of cells treated with DMSO 1%, while absorbance sample is the absorbance of cells treated with the test sample.

The results of this research were tested using a non-parametric test, Kruskal–Wallis test followed by Mann–Whitney.

## Results

### The phytochemical screening of fig leaf extract

The phytochemical screening of fig leaf extract showed positive results on flavonoids, terpenoids, and tannins but negative results on saponin and alkaloid.

### Antibacterial activity of fig leaf extracts

**Figure-1:**
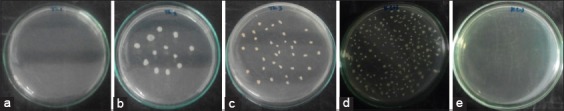
The result of inoculating of *Enterococcus faecalis* bacteria onto nutrient agar media with fig leaf extract (*Ficus carica* Linn.) at concentration of (a) 50%, (b) 37.5%, (c) 25%, (d) cell control, (e) media control.

The results of this research showed that fig leaf extract at a concentration of 25% was considered as MIC, while MBC was at a concentration of 50%. Consequently, further research was conducted to determine the accuracy of MBC using a concentration between 50% and 25% that is 37.5%. There was no *E. faecalis* growth at 50% concentration of fig leaf extract, but at a concentration of 37.5% and 25%, *E. faecalis* still grows (Figure-1). fig leaf extract at 37.5% concentration showed that the number of *E. faecalis* colonies was lower than at 25% concentration. Hence, the concentration of 37.5% was considered as MIC. Therefore, cytotoxicity test was performed at 37.5% and 50% concentration. The mean and standard deviation of *E. faecalis* are presented in Table-1.

**Table-1:** Mean and standard deviation of *Enterococcus faecalis* colony.

Group	n	Mean	SD
a - 50%	3	0	0
b - 37.5%	3	13,0000^a^	2,00000
c - 25%	3	28,0000^b^	9,53939
d - cell control	3	139,3333^c^	34,67468
e - media control	3	0	0

Superscript with different letters in the same column show significant difference (p<0.05). SD=Standard deviation

### Cytotoxicity activity of the extracts on fibroblast cell culture

The results showed that the fig leaf extract at the concentration of 37.5% had 97.6% cell viability, however, at higher concentration (50%) had 77.7% cell viability. This study showed no toxicity at the concentration tested on fibroblast cell culture. The mean and standard deviation of OD are presented in Table-2. The higher the OD value, the higher the viability of the cell.

**Table-2:** Mean and standard deviation of MTT assay.

Concentration	n	Mean (OD)	Cell viability (%)	SD	P
37.5%	10	0.5516^a^	97.6	0.01386	0.000*
50%	10	0.4394^b^	77.7	0.02036	
Cell control	10	0.5650^c^		0.03708	

Superscript with different letters in the same column showed significant difference (p<0.05). SD=Standard deviation, MTT=3-(4,5-dimethylthiazol-2-yl)-2,5-diphenyltetrazolium bromide, OD=Optical density

## Discussion

### Antibacterial activity of fig leaf extracts

This study showed that fig leaf extracts had MIC and MBC for *E. faecalis* at concentrations of 37.5% and 50%, respectively, and it is presented in Table-1. It proves that fig leaf extracts had antibacterial activity. Some studies that used herbal extracts as antibacterials suggest that the antibacterial activity is due to the content of flavonoids, tannins, and terpenoids. Based on the phytochemical screening of fig leaf extracts in this study, it contains the same active ingredients, namely, flavonoids, tannins, and terpenoids. These active compounds have their own mechanism [7].Considering the presence of compounds such as flavonoids, tannins, and terpenoid, which proven to have antimicrobial activity, it is concluded that the antimicrobial activity of the extracts may be related to the presence of these compounds. Regarding the MBC, the value was 50%.

Antibacterial mechanisms of flavonoids were to inhibit nucleic acid synthesis, inhibit energy metabolism and disrupt cytoplasmic membrane function [21]. The similar research showed that there was a correlation between antibacterial activity and membrane interference by reducing membrane fluidity of bacterial cells [22]. Liposomes in bacterial membranes due to the fig leaf containing flavonoids can indicate leakage of small molecules from the intraliposomal space and aggregation of liposomes, leading to bacterial membrane damage [21].

Flavonoids as antibacterial have multiple cellular targets. Flavonoids can form complex with proteins through non-specific forces, such as hydrogen bond and hydrophobic effects, as well as by forming covalent bond formation. Thus, their antimicrobial mode of action may be related to the ability to inactivate microbial adhesins, enzymes, cell envelope transport proteins, and so forth. Lipophilic flavonoids also have the ability to disrupt microbial membranes [23]. Previous research showed that flavonoids may be interfering with energy metabolism since energy is inhibited strongly in oxygen consumption in bacteria [24].

Moreover, tannins considered as a toxic compound for bacteria can bind their cell walls as well as prevent their growth and protease activity [25]. Tannins can inhibit the growth of bacteria by forming hydrogen bonds with proteins in bacterial cells resulting in protein denaturation so that bacterial metabolism was became impaired. The formation of hydrogen bonds between tannins and proteins can lead to changes in the shape of protein molecules that can decrease their biochemical activity. Tannins can also inhibit the growth of bacteria as well as kill them by reacting with phospholipids contained in the cell membrane of bacteria, triggering membrane damage and important metabolite leakage, resulting in an inactive bacterial enzyme system, causing growth inhibition and bacterial death [26]. Meanwhile, terpenoid compound is also known to be active against bacteria. The antibacterial activity of terpenoids is thought to involve membrane disruption triggered by the lipophilic compounds [25].

Flavonoids, tannins, and terpenoid compounds in fig leaves with their respective mechanism, furthermore, work synergistically against *E. faecalis*. This mechanism can trigger the physiological activity of the *E. faecalis* to decrease, causing *E. faecalis* growth to be inhibited and *E. faecalis* death. *E. faecalis* is the most resistant bacteria in the root canal as well as one of the causes of root canal failure.

fig leaf extract with a MBC value of 50% for *E. faecalis* then will be used as a root canal irrigation material candidate. The cytotoxicity test should be performed to comply the prescribed requirements, one of which is compatible ingredients for dentistry because some compounds of plants are toxic to our normal system; therefore, safety is critical in the development of novel drugs [15].

### Cytotoxicity activity of the extracts on fibroblast cell culture

Cytotoxicity in cell culture is typically expressed as LC_50_, which means that the concentration of a given agent is lethal to 50% of the cells. Moreover, the most common way to describe cytotoxicity in cell culture is LC_50_, the concentration of a drug that kills half of the tested cells in culture [27].

In this research, the cytotoxic effects of the fig leaf extract at the concentrations of 37.5% and 50% were examined on human gingival fibroblast using MTT assay. The results showed that the fig leaf extract has no cytotoxic effect. Percentage cell viability at MIC (37.5%) was 97.6% while at MBC (50%) was 77.7% after the 24 h exposure (Tabel-2). Similarly, the results of a previous research showed that cell viability decreased as the concentration of the plant extract increased [28], but it had no cytotoxic effect since cell viability obtained was >50% [27].

fig leaf extract at 50% concentration was not cytotoxic but is capable of killing *E. faecalis*, so it has the potential to be further investigated as a root canal irrigation material candidate. Further studies will be designed to investigate its biomedical applications with a detailed mechanism through appropriate experimental models.

## Conclusion

It can be concluded that fig leaf extract at 50% concentration has antibacterial activity against *E. faecalis* and not toxic to fibroblast cells.

## Authors’ Contributions

IN has designed the plan of research work and a research coordinator. RDL and DPB performed data collection. DR and RHS participated in the laboratory work and analyzed the results. All authors read and approved the final manuscript.
